# Work-Family Enrichment: Influence of Job Autonomy on Job Satisfaction of Knowledge Employees

**DOI:** 10.3389/fpsyg.2021.726550

**Published:** 2021-12-02

**Authors:** Song Jing, Zhuoyu Li, Daniel M. J. J. Stanley, Xia Guo, Wang Wenjing

**Affiliations:** ^1^School of Public Administration, Southwestern University of Finance and Economics, Chengdu, China; ^2^Sichuan Vocational and Technical College of Communications, Chengdu, China; ^3^School of Finance, Southwestern University of Finance and Economics, Chengdu, China

**Keywords:** satisfaction, knowledge employees, work-family enrichment, self-efficacy, job autonomy, conservation of resources theory

## Abstract

In order to explore the specific path of the influence of job autonomy on the satisfaction of knowledge employees, the current study deduced and established a Chain Mediation Model, which was based on the Resource-Gain-Development Model and the Conservation of Resources Theory. Primary data were gathered through questionnaire surveys at several cities in China by using a professional platform named “Wenjuanxing.” The target populations were employees with a bachelor’s degree or above, who were engaged in higher knowledge content and have mastered certain professional knowledge and skills, including technical R & D personnel, management personnel, professionals (such as accountants, lawyers, and medics) and other personnel generally recognized by the academic community. In order to improve the reliability of the sample and reduce the error caused by regional differences, the questionnaires were disseminated to involve as many cities in China as possible, such as Tianjin, Beijing, Chengdu, Wuhan, and Guangzhou. SPSS24.0 and Aoms24.0 were used as multivariate data analysis tools for statistical analysis. The results showed that job autonomy can significantly improve the satisfaction of knowledge employees; however, it cannot affect the satisfaction of knowledge employees through self-efficacy. The findings of the study also revealed that job autonomy has a positive impact on both resource source domain satisfaction and resource acceptance domain satisfaction through work-family enrichment, especially the positive emotions in the resource source domain. Job autonomy improves the self-efficacy of knowledge employees, which, in turn, improves their overall satisfaction through the work-family enrichment path.

## Introduction

Drucker (1999), a renowned master of management, was the first to realize that employees with the ability to apply knowledge can create greater value for the organization. Afterward, some scholars called the employees “knowledge employees,” who have received more education and worked mainly mentally, such as management personnel, R & D personnel, management consultants, and professionals (Zhao, 2000; Kuai et al., 2019). In recent years, there have been considerable and rapid changes in economic development, industrial transformation, and aging. These changes have put enormous pressures on knowledge employees, both within work and family domains, which then deteriorated their satisfaction and, in turn, impacted and affected their work performance. Modern times have seen an increase of contributions of knowledge employees to their enterprises. Enterprises themselves are, therefore, faced with the never-ending task of finding ways to improve the satisfaction and stability of their knowledge employees in order to achieve higher performance levels and to realize their maximum capabilities. This has become a problem that the enterprises have placed emphasis on, and have paid special attention to, a problem that they urgently need to solve.

Knowledge employees have received more education, acquired more knowledge and skill set, and have strong autonomy needs. The satisfaction of knowledge employees with strong autonomy is greatly affected by environmental changes. In recent years, and due to the intensification of aging, both husbands and wives equally need to bear the pressure of taking care of the upper and lower generations in the family. In terms of work, and due to improved education levels and increased stress in life, many women have opted to get jobs, thus forming more dual-career family models (Harris et al., 2015). Therefore, meeting the autonomy needs and improving the work family relationship of knowledge employees have become important factors in improving employee satisfaction. Relevant studies have confirmed this. For example, Luthans and other studies (Luthans et al., 2007) have proved that employees’ perception of job autonomy will effectively improve psychological experiences and psychological states of employees (Luthans et al., 2007). Butler et al. found that job autonomy can not only effectively improve the work-family enrichment but also effectively determine job satisfaction of employees (Butler et al., 2005; Haar and Spell, 2009; Baral and Bhargava, 2011; Matijaš et al., 2018).

However, the path of job autonomy affecting satisfaction of knowledge employees needs to be elaborated. Job autonomy is an important work resource for knowledge employees (Yang and Sun, 2011), while non-hardware resources provided by organizations are related to individual perception evaluation (Yang et al., 2019; Gadeyne et al., 2018). Self-efficacy of employees is cognition and evaluation of employees of the environment and its resources (Bandura et al., 1999). However, there is a lack of research on the relationship between job autonomy and self-efficacy of employees. Previous studies have shown that one of the important predictors of work-family enrichment and satisfaction is job autonomy, but no study has connected the four with self-efficacy (job autonomy – self-efficacy – work-family enrichment – satisfaction). In addition, some scholars believe that enrichment can only have a positive impact on the contribution domain, that is, work to family enrichment can only positively predict job satisfaction, while family to work enrichment is only related to family satisfaction (Carlson et al., 2014; Vadivukkarasi and Ganesan, 2015). Some scholars believe that enrichment can have a positive impact on both contribution and acceptance domains (McNall et al., 2010). Wayne et al. (2007) studied the antecedents, results, and regulatory variables of work-family promotion through the integration of Conservation of Resources (COR) theory and other related theories. However, Wayne and his successors did not verify whether the contribution domain of resources is more closely related to the contribution domain or the acceptance domain through work-family enrichment (Wayne et al., 2004, 2006, 2007). Therefore, combined with the psychological capital in positive psychology, this study selects self-efficacy as the intermediary variable, attempts to use COR and RGD theory to study the impact of job autonomy on improving the satisfaction of knowledge employees from the perspective of work-family enrichment, and further clarifies the field influence relationship between the antecedents and outcome variables of work-family enrichment by comparing whether the contribution domain has a greater impact on the contribution domain through work-family enrichment (Model 1), or whether the contribution domain has a stronger impact on the acceptance domain through work-family enrichment (Model 2).

### Theoretical Foundation and Hypotheses Development

The hypotheses in this study are based on the Conservation of Resources Theory (COR) (Hobfoll et al., 2018) and the Resource Gain Development Perspective (RGD) (Wayne et al., 2007). The basic view of the COR model is that people strive to retain, protect, and build resources, and worry about the potential or actual loss of resources. The premise of RGD is that individuals have the motivation tendency to maximize growth, development, and related potential. When they invest in a role, they will obtain growth resources; when it maximizes the use of resources, it can obtain positive benefits. Based on the theories of COR and RGD, it can be found that the views of scholars can be summarized as Motivation-Resource-Gain-Enrichment. The basis of enrichment is that individuals have the motivation to obtain resources. The increase of initial resources will further bring more resource benefits and accumulation so as to enter the income spiral. Based on the ideas of COR and RGD, knowledge employees tend to grow, develop, and pursue the maximization of relevant potential. Therefore, knowledge employees have the motivation to obtain and preserve resources in the field of work and family. At the same time, according to the COR model, although the loss of resource is stressful, individuals may use other resources to offset the net loss. On the premise of limited total resources in the field of work and family, the resources provided by the environment and the differences in individual pursuit and allocation of resources have become important factors affecting work, family, and work-family promotion.

### Job Autonomy, Job Satisfaction, and Family Satisfaction of Knowledge Employees

Enterprises are in the era of knowledge economy, and knowledge is an important resource for their survival and development. Employing knowledge workers and effectively absorbing the spillover effect of knowledge can help enterprises obtain and maintain competitive advantage. Enterprises rely more and more on the ability of knowledge employees to quickly solve professional and technical problems, and use their unique knowledge socialization to enhance the core competitiveness of the organization (Stenius et al., 2017), thus making it very important for enterprises to retain and motivate knowledge employees.

The empirical study found that if individuals have strong job satisfaction, they will work harder to improve performance (Wayne et al., 2004), which will help enterprises to strengthen and maintain the core competitiveness of the organization. Satisfaction can reflect the overall attitude of knowledge employees toward the field and also represents their emotional states. Therefore, how to understand and improve the satisfaction of knowledge employees should become the focus of enterprises. The most important domain of employees is the domain of work and family. Therefore, this study chooses job satisfaction and family satisfaction to reflect the overall satisfaction of knowledge employees.

As a kind of work resource, job autonomy will directly affect individual job satisfaction. On the one hand, due to the novel and challenging nature of work of knowledge employees, they have the work motivation that requires a high degree of freedom and decision-making power to deal with complex work (Jiang and Zhao, 2001); On the other hand, job autonomy represents an important emotional resource for the trust, support, and respect for employees of the organization (Kim et al., 2019). According to the theory of COR and RGD, when employees obtain important resources, they will strive to preserve and utilize them to maximize their value. Job autonomy reflects the trust between work organizations and individuals. Knowledge employees will strive to use these resources to deal with complex work that requires a high degree of freedom. Through the effective completion of work, job satisfaction of employees will be improved.

The COR theory indicates that the role of resources promoting positive emotions is universal (Seibert et al., 2001; Wayne et al., 2007), because the state of poor resources will bring more stress and anxiety to employees, and the situation of abundant resources makes people feel satisfied and safe (Hobfoll, 2011; Hobfoll et al., 2018). The autonomy of jobs will liberate the stress and anxiety of nervous knowledge employees and promote their positive emotions. Relaxed employees with positive emotions are bound to release more emotions in the family domain and promote the improvement of family satisfaction (Bhowmick and Mulla, 2016). So, in light of this, the study proposes the following hypotheses:

**Hypothesis 1:** The job autonomy of knowledge employees is positively correlated with job satisfaction and family satisfaction.
**Hypothesis 1-1:** The job autonomy of knowledge employees is positively correlated with job satisfaction.
**Hypothesis 1-2:** The job autonomy of knowledge employees is positively correlated with family satisfaction.

### The Mediating Role of Self-Efficacy

The self-efficacy of knowledge employees is a psychological resource that cannot be ignored in workforce management. From the COR and RGD theory, we can deduce that people with more original resources are more likely to accumulate resources to cope with the pressure of various complex environments. The resources obtained from the environment can be transformed into the psychological resources possessed by individuals (Yu et al., 2015). Empirical research shows that, in a series of transformations of individuals, work, and family, employees rely on the supportive situation resources gained in the work field to enhance their psychological resources (Zhang et al., 2015). Generally speaking, resources include environmental resources and individual resources. In the current study, environmental resources refer to work autonomy, and personal resources refer to self-efficacy. Employees with high job autonomy have the characteristics of high self-adjusting ability and can often quickly adapt to the environment so as to achieve the set goals and projected results, thereby enhancing their sense of self-efficacy (Wang and Zhao, 2011). Therefore, we speculate that job autonomy may positively enhance self-efficacy.

On the one hand, individuals with high self-efficacy have a positive impact on job satisfaction through their achievements in work. Luthans et al. (2007) first evaluated the four dimensions of psychological capital (resilience, optimism, self-efficacy, and hope) and studied the relationship between these four dimensions and job satisfaction. The results pointed out that self-efficacy is positively correlated with job satisfaction. On the other hand, high self-efficacy will also be reflected in the family domain. According to the COR theory, resources are considered to be an important part of ability of people to resist pressure. The more resources provided by the work environment, the higher the level of self-efficacy of knowledge employees. The self-efficacy of personal resources has the ability to transfer sensitively between work and family domains. It helps employees cope with the pressure of work and family at any time, and makes up for the resource consumption across domains. At the same time, self-efficacy provides additional resources, which is conducive to the completion of tasks in work and family so as to improve the satisfaction of the two domains. Based on this, the current study assumes that:

**Hypothesis 2:** The job autonomy of knowledge employees has a positive impact on job satisfaction and family satisfaction through self-efficacy.
**Hypothesis 2-1:** The job autonomy of knowledge employees has a positive impact on job satisfaction through self-efficacy.
**Hypothesis 2-2:** The job autonomy of knowledge employees has a positive impact on family satisfaction through self-efficacy.

### The Mediating Role of Work-Family Enrichment

Work-family enrichment is to study the relationship between resources and outcome variables in various domains by a positive perspective. Based on the COR theory, the RGD model comprehensively analyzes the antecedent variables (resources) and outcome variables of work-family enrichment function. It is a mature model in the field of work-family enrichment research. Therefore, this study chooses the RGD model to explore the internal mechanism between job autonomy and job satisfaction.

The RGD model explains that the more work resources, the higher the work to family enrichment level, then the job autonomy as a work resource can effectively improve the work to family enrichment. Job autonomy means having freedom in work and responsibilities, being able to flexibly arrange time, thus improving work efficiency, reducing the chance of conflict between work and family needs (Zhang et al., 2010), making it possible for the two domains to gain from each other (Baral and Bhargava, 2011; Kiburz et al., 2017).

The dimensions of outcome variables of the RGD model involve the family domain and the work domain. If the resources obtained from work can help individuals better complete family affairs, and individuals can give more feedback to those who provide resources (Wayne et al., 2006), and then work to family enrichment will have an impact on the outcome variables of the work domain. Relevant empirical studies also showed that the higher the level of work to family enrichment, the higher the job satisfaction will be (Tang et al., 2014; Daniel and Sonnentag, 2016). Furthermore, the high level of work-family enrichment of employees can improve their levels of individual mental health, which is conducive to improving their work performance, which then boosts their morale and resulting in good moods. Positive emotions of employees can help establish a happy family, create a harmonious family atmosphere, and encourage them to be more willing to bear the responsibility of raising the next generation. All these can effectively improve marriage quality and family satisfaction of employees (Voydanoff, 2004). The interpersonal communication ability and knowledge that knowledge employees have in their work can be applied to the family field by promoting their relationships with their families and improving the efficiency of their work, thus improving family satisfaction.

Resource spillover effect in COR theory can explain the principle of cross-domain resource conversion. Resources between domains can transgress and overflow each other and play a role in the domain of each other (Amstad et al., 2011). As a kind of work resource, job autonomy accumulates constantly in the domain of work. The positive behaviors and high emotions will permeate into the family domain and promote the improvement of family relationship. However, Carlson et al. (2006) believed that, when the resources acquired in the work domain enhance the function of the individual in the family domain, the individual recognizes the source of income, and thus has a greater satisfaction with the domain providing the income. The former agrees with cross-domain enrichment, while the latter agrees with contribution domain enrichment. The existing empirical research results are also inconsistent. Some research results show that job autonomy is positively correlated with job satisfaction and family satisfaction through the path of work to family enrichment (Zhang et al., 2012). Some studies only support one of them and deny the other. In order to compare the effect of job autonomy on job satisfaction (contribution domain) and family satisfaction (acceptance domain) through work-family enrichment, the current study posits the following hypotheses:

**Hypothesis 3:** The job autonomy of knowledge employees has a positive effect on job satisfaction and family satisfaction through work-family enrichment.
**Hypothesis 3-1:** The job autonomy of knowledge employees has a positive effect on job satisfaction through work-family enrichment.
**Hypothesis 3-2:** The job autonomy of knowledge employees has a positive effect on family satisfaction through work-family enrichment.

### The Chain Mediating Role of Self-Efficacy and Work-Family Enrichment

At the same time, existing studies have shown that there is a correlation between self-efficacy and work-family enrichment (Yang et al., 2012).

Combining the COR and RGD theory, individuals have the motivation to preserve resources in the work and family domain, and, when they invest in a role, they have the tendency to acquire, utilize, grow, and develop resources. The increase of resources will further bring more resource benefits and accumulation. As an important work resource, high job autonomy means that employees obtain more work environment resources from the organization. Thus, employees can quickly adapt to the work environment, complete work goals, and enhance their sense of self-efficacy. This transformation from work environment resources to personal resources, according to RGD, can be permeated and extended to the family domain to accumulate across the border. The positive psychological state is the accumulation of energy for family roles, helping to improve the behavior performance of individuals in cross-role fields, and individuals will get more work to family enrichment. The self-efficacy gained in work improves the confidences of employees, which is of great benefit for employees to shoulder more responsibilities, invest more efforts, complete challenging tasks in the family domain, and enhance the level of work-family enrichment from all aspects. To be specific, knowledge employees put more efforts into housework and emotional communication with family members and company due to higher self-efficacy. With the continuous improvement of performance in family, family members tend to improve the praise of knowledge employees so as to realize the work to family enrichment. Many empirical studies also show that self-efficacy, as one of the psychological resources, is positively correlated with work-family enrichment (Siu et al., 2010; Yang et al., 2012; Premchandran et al., 2019). Therefore, this study establishes a chain mediating model to explore whether self-efficacy and work-family enrichment play a chain mediating role between job autonomy and job satisfaction, as well as job autonomy and family satisfaction. Thus, the following hypotheses are proposed:

**Hypothesis 4:** The job autonomy of knowledge employees has a positive effect on job satisfaction and family satisfaction through the chain-mediating effect of self-efficacy and work-family enrichment.
**Hypothesis 4-1:** The job autonomy of knowledge employees has a positive effect on job satisfaction through the chain-mediating effect of self-efficacy and work-family enrichment.
**Hypothesis 4-2:** The job autonomy of knowledge employees has a positive effect on family satisfaction through the chain-mediating effect of self-efficacy and work-family enrichment.

## Materials and Methods

### Participants

This study selected employees with a bachelor’s degree or above, who were engaged in higher knowledge content and have mastered certain professional knowledge and skills as the sample of knowledge employees, including technical R & D personnel, management personnel, professionals (such as accountants, lawyers, and medics), and other personnel generally recognized by the academic community (Kuai et al., 2019). In order to improve the reliability of the sample and reduce the error caused by regional differences, these questionnaires were disseminated to involve Tianjin, Beijing, Chengdu, Wuhan, Guangzhou, and other regions as much as possible.

In this study, the respondents were employees from program development enterprises, law firms, accounting firms, and universities. After obtaining the support of department leaders, the questionnaires were distributed during lunch break and were to be completed before punching out and signing off from the day’s work. By using a professional platform named “Wenjuanxing,” the questionnaires were distributed to the employees through the internal e-mail of the company. All employees completed the questionnaires anonymously. A total of 406 questionnaires were collected, of which some were eliminated due to the regularly repeated responses, less than 100 seconds’ response time, and also those with junior college degrees or below; therefore, our final sample size was 305, with an effective valid response rate of 75.12%. The average age of the respondents was 28.46 years old, including 171 males (56.07%) and 134 females (43.93%); for the education level, undergraduates account for 75.41%, postgraduates and above personnel accounted for 24.59%; the average working year was 4.73. There were 58 people from the computer/Internet industry, 36 from the accounting/financial industry, 23 from the law industry, and 51 from the university.

### Measures

In the current study, the mature measurement tools widely used locally and abroad were also used, and, on the whole, the Likert five-point scoring method was adopted.

Job autonomy scale, compiled by Breaugh (1985), was used in the study. The scale was first used to measure the dimension of job autonomy in the work characteristics scale, and it used five levels to measure the level of job autonomy. The scale includes nine items and was divided into three dimensions: method autonomy, arrangement autonomy, and standard autonomy. For example, “I can decide which method to use to complete my work,” “I can choose my work procedure,” and so on. The reliability of the scale was analyzed by the scholar, and the internal consistency coefficient was 0.78.

General self-efficacy scale (GSES) was used to measure self-efficacy, which was first completed by Professor Schwarzer, a German clinical and health psychologist, and his colleagues in 1981. The questionnaire of the scale was reduced from 20 items to 10 items. At present, the scale has been translated into at least 25 different languages and has been widely used in international empirical research. The Chinese version of GSES used in the study was first compiled by Zhang and Schwarzer (1995). Up to now, GSES in Chinese has been proved to have good reliability and validity (Shen and Tang, 2004). There are 10 items in the scale. The respondents can choose 1–5 scores according to their own conditions. The higher the scores indicate the higher the general self-efficacy of the respondents, such as “if I try to do it, I can always solve the problem.” The internal consistency coefficient of the scale was between 0.75 and 0.91, which means a good goodness of fit.

Work-family enrichment scale in line with localization of China was first compiled by Chinese scholar Tang et al. (2009). The scale refers to the questionnaire and theoretical research results of foreign scholars, and is compiled on the basis of the investigation of eight enterprises in China. The scale includes work to family enrichment and family to work enrichment. In this study, only the work to family enrichment was involved, so the items of this part in the scale were extracted. The internal consistency coefficient of work to family enrichment scale was 0.86, with a total of seven items, such as “work makes me learn to listen and understand different views from myself so that I can perform better in getting along with my family” and so on. The respondents can choose from five levels according to the situation, and the high scores indicate that the work to family enrichment level of the respondents is high.

Job satisfaction scale was the revised short version of Minnesota by Zhang and Gu (2010), with a total of 18 questions, such as “have the opportunity to deal with things in their own way,” “satisfied with the way the superior makes decisions,” and so on. It is divided into three dimensions: situational factors, outcome factors, and personal factors. The internal consistency of each dimension was 0.91, 0.82, and 0.65, respectively. The respondents can choose from five levels according to the situation, and the high scores indicate the high level of job satisfaction.

Satisfaction with family life scale (SWFL) developed by Zabriskie and McCormick (2003) was used to measure the satisfaction of respondents with their family life. SWFL comes from the revision of satisfaction with life scale (SWLS), in which the word “family life” replaces the word “life” in the original project. SWFL consists of five projects, such as “my family life is close to ideal in many ways,” “my family living conditions are very good,” etc. The scale has good psychometric characteristics, including structural validity, internal consistency (a = 0.93), and test-retest reliability (*r* = 0.89). The respondents can choose from five levels according to the situation, and the high scores indicate that the respondents have a high level of family satisfaction.

## Results

SPSS24.0 software was used for reliability analysis, common method deviation analysis, descriptive statistical analysis, and correlation analysis. Amos24.0 software was used to analyze the discriminant validity of the model and establish the structural equation model. Due to the chain intermediary model seriously violating the normal distribution hypothesis and the limited number of samples in this study, it was not suitable to use the Sobel test method based on the normal hypothesis and large sample premise. In addition, Preacher et al. pointed out that the bootstrap method does not need the normal distribution hypothesis, nor does it need the standard deviation to estimate the mediating effect interval (Preacher and Hayes, 2008). As long as the mediating effect interval does not include 0, it means that the effect is significant. Therefore, this study chose the bootstrap method recommended by Preacher et al. to test the chain-mediating effect, fitting the relationship between latent variables and testing the mediating effect.

### Common Method Deviation Test

The data of all variables in this study were collected by questionnaires in the workplace, which easily leads to the homogenization of data sources and raters, the consistency of measurement, project context and project itself, and the artificial covariation between predictive variables and calibration variables. Artificial covariance is one of the systematic errors, which can seriously confuse the research results and mislead the research conclusions. The common method deviation can reflect the severity of the systematic error of the measurement results, so it is necessary to test the common method deviation of the questionnaires before analysis. Using Harman’s single factor test, all questionnaire items were analyzed by factor analysis without rotation. In the results, the variance of the first principal component was 39.09, less than 40%, and the number of principal components with eigen values greater than 1 was 8 (greater than 2). This means that there was no common method bias effect between the measured variables.

### Reliability Analysis of Research Tools

This study used the reliability analysis of SPSS24.0 to test the reliability of job autonomy scale, self-efficacy scale, work-family enrichment scale, job satisfaction scale, and family satisfaction scale. As shown in Table 1, the internal consistency coefficients of the scales in this study were all above 0.85, indicating that each scale had good reliability.

**TABLE 1:** Mean value, standard deviation, and correlation coefficient of each variable.

Variable	M	SD	Cronbach coefficient	1	2	3	4	5
Job autonomy	3.67	0.77	0.9	1				
Self-efficacy	3.65	0.66	0.91	0.65**	1			
Work to family enrichment	3.89	0.75	0.9	0.5**	0.54**	1		
Job satisfaction	3.76	0.7	0.95	0.66**	0.6**	0.73**	1	
Family satisfaction	3.6	0.85	0.88	0.48**	0.45**	0.55**	0.59**	1

N = 305, *p < 0.05, **p < 0.01, ***p < 0.001.

### Confirmatory Factor Analysis

In order to verify the validity of job autonomy, self-efficacy, work to family enrichment, job satisfaction and family satisfaction, and the corresponding measurement parameters of each scale, confirmatory factor analysis (CFA) was conducted. Due to the large number of items, the items of the five scales were first analyzed by factor analysis and then packaged.

According to the results of confirmatory factor analysis in Table 2, the results of the five-factor model composed of job autonomy, self-efficacy, work to family enrichment, job satisfaction, and family satisfaction were: X2/df = 2.23 < 3, RMSEA = 0.06 < 0.08, CFI = 0.98 > 0.9, TLI = 0.97 > 0.9. In other model combinations, not all of them fit, so the five-factor model was the best and had better discriminative validity.

**TABLE 2:** Results of confirmatory factor analysis.

Model	X^2^	df	X^2^/df	RMSEA	CFI	TLI
Five-factor model	98.02	44	2.23	0.06	0.98	0.97
Four-factor model	250	48	5.21	0.12	0.93	0.91
Three-factor model	445.98	51	8.75	0.16	0.87	0.83
Two-factor model	637.7	53	12.03	0.19	0.8	0.75
Singel-factor model	890.2	54	16.49	0.23	0.71	0.65

*N = 305.*

### Descriptive Statistical Analysis and Correlation Analysis

The mean value, standard deviation, and correlation matrix of the main variables are listed in Table 1. The results showed that there were significant positive correlations among the five variables. Specifically, job autonomy was positively correlated with self-efficacy (*r* = 0.65, *p* < 0.01), work to family enrichment (*r* = 0.5, *p* < 0.01), job satisfaction (*r* = 0.66, *p* < 0.01), and family satisfaction (*r* = 0.48, *p* < 0.01); self-efficacy was positively correlated with work to family enrichment (*r* = 0.54, *p* < 0.01), job satisfaction (*r* = 0.6, *p* < 0.01) and family satisfaction (*r* = 0.45, *p* < 0.01); work to family enrichment was positively correlated with job satisfaction (*r* = 0.73, *p* < 0.01) and family satisfaction (*r* = 0.55, *p* < 0.01); there was a significant positive correlation between job satisfaction and family satisfaction (*r* = 0.59, *p* < 0.01).

### Structural Equation Model Analysis

Fang et al. (2014) said that the establishment of the structural equation model for chain-mediating analysis does not only deal with explicit variables and latent variables at the same time but can also analyze the relationship of multiple mediating variables at once, which was suitable for the current study that there were two mediating variables. Therefore, based on hypothesis Model 1, hypothesis Model 2, and the analysis of the relationship between variables, this study established a chain intermediary structural equation model with latent variables to explore the mediating effects of job autonomy, self-efficacy, work to family enrichment, job satisfaction, and family satisfaction.

Wu and Wen (2011) pointed out that each dimension can be packaged as an indicator according to the inner dimension of latent variables. If the model is complex and the sample size is small, each dimension can be packaged as an indicator. Due to the complexity of the model and the limited sample size, the variable dimension packing method was adopted. The average score of each dimension in the variable was taken, and the packaged observation variable was replaced by the average score of each item in the package. It was found that the initial fitting index of Model 1 was: X2 = 693.62, X2/df = 3.10, CFI = 0.90, IFI = 0.90, RMSEA = 0.08, having a good fitting effect.

**FIGURE 1:**
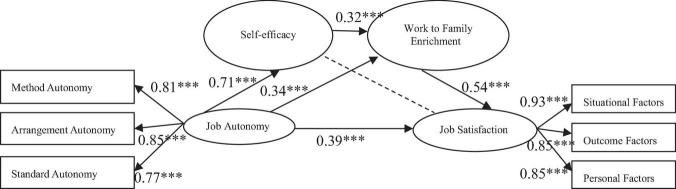
The chain mediating-effect model of job autonomy and job satisfaction. The dotted line indicates the path deleted during model modification because the path coefficient is not significant.

The results of Model 1 proved that job autonomy can directly predict job satisfaction (β = 0.39, *p* < 0.001), Hypothesis 1-1 was supported; the path coefficient of job autonomy and self-efficacy (β = 0.71, *p* < 0.001) and work to family enrichment (β = 0.34, *p* < 0.001) was significant; the path coefficient of self-efficacy and job satisfaction was not significant (β = 0.06, *P* = 0.31), Hypothesis 2-1 was not supported. Since the path coefficient of self-efficacy and job satisfaction was not significant, this study used the principle of eliminating the nonsignificant path in the order of standardized coefficient “from small to large” and obtains the modified model, as shown in Figure 1. The fitting index of modified Model 1 was: X2 = 694.65, X2/df = 3.09, CFI = 0.90, IFI = 0.90, RMSEA = 0.08.

**FIGURE 2:**
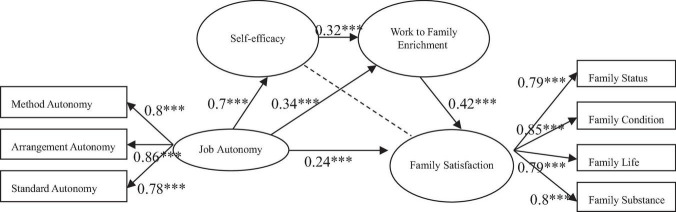
The chain-mediating effect model of job autonomy and family satisfaction. The dotted line indicates the path deleted during model modification because the path coefficient is not significant.

The initial fitting index of Model 2 was: X2 = 707.79, X2/df = 2.88, CFI = 0.90, IFI = 0.90, RMSEA = 0.08. The results of Model 2 showed that job autonomy had a significant direct predictive effect on family satisfaction (β= 0.24, *p* < 0.01), Hypothesis 1-2 was, therefore, supported; the path coefficient of job autonomy and self-efficacy (β= 0.7, *p* < 0.001) and work to family enrichment (β= 0.34, *p* < 0.001) is significant; the path coefficient of self-efficacy and family satisfaction was not significant (β= 0.11, *p* = 0.19); thus, Hypothesis 2-2 was not supported. Similarly, because the path coefficient of self-efficacy and family satisfaction was not significant, this study removed the nonsignificant path in the order of standardized coefficient “from small to large” and obtained the revised Model 2, as shown in Figure 2. The modified model fitting index was: X2 = 709.48, X2/df = 2.87, CFI = 0.90, IFI = 0.90, RMSEA = 0.08.

### Bootstrap Mediation Analysis

Taylor et al. (2008) summarized and compared the test methods of multiple mediations, including causal stepwise regression, the test of the significance of variable coefficients, and the bootstrap method. Through empirical research, it was found that the bootstrap method had the best effect, which was also supported by Fang et al. (2014). Therefore, the current study used the bootstrap program to test the significance of mediating effect. Repeated random sampling of 2,000 from the original data (*N* = 305) as bootstrap samples, modified Model 1, and modified Model 2 was fitted, and then the estimated values of 2,000 mediating effects were generated and saved to form an approximate sampling distribution. At the same time, the average path values of mediating effects were calculated, and these effect values were sorted according to the numerical value. The 95% confidence interval of mediating effects was estimated by the 2.5th percentile and 97.5th percentile. If the 95% confidence interval of these path coefficients does not include 0, the mediating effect is significant. Figure 1 showed that the path coefficient of self-efficacy and job satisfaction was not significant, so the influence of job autonomy on job satisfaction was realized through the other two intermediary paths and direct paths. It can be seen from Table 3 that the results of the modified Model 1 indicated that the two confidence intervals of “job autonomy→work to family enrichment→job satisfaction” (0.06, 0.31) and “job autonomy → self-efficacy → work to family enrichment → job satisfaction” (0.05, 0.22) do not include 0, and the indirect effect values were also within the confidence interval, showing a significant mediating effect. That is to say the mediating effect of “job autonomy → work to family enrichment → job satisfaction” and the chain-mediating effect of “job autonomy → self-efficacy → work to family enrichment → job satisfaction” was significant. Thus, Hypothesis 3-1 and Hypothesis 4-1 are supported.

**TABLE 3:** Path analysis of the effect of job autonomy on job satisfaction.

Path relationship		Indirect effect of standardization	95% confidence interval	
			Lowerbound	Upperbound
Direct effect	JA→JS	0.44	0.28	0.56
Mediating effect	JA→WFE→JS	0.34 × 0.55 = 0.19	0.06	0.31
	JA→SE→WFE→JS	0.71 × 0.32 × 0.55 = 0.12	0.05	0.22
Total mediating effect		0.34 × 0.55 + 0.71 × 0.32 × 0.55 = 0.31	0.22	0.41
Total effect		0.44 + 0.31 = 0.75	0.57	0.87

*JA, job autonomy; WFE, work to family enrichment; JS, job satisfaction; SE, self-efficacy.*

Figure 2 showed that the path coefficient of self-efficacy and family satisfaction was not significant, so the influence of job autonomy on family satisfaction was realized through the other two intermediary paths and direct paths. It can be seen from Table 4 that the results of the modified Model 2 indicated that, in the two confidence intervals of the “job autonomy → work to family enrichment → family satisfaction” path (0.05, 0.37) and the “job autonomy → self-efficacy → work to family enrichment → family satisfaction” (0.05, 0.25) path, 0 was not included, and the indirect effect values were within the confidence interval, showing that the mediating effect was significant. That is to say the mediating effect of “job autonomy → work to family enrichment → family satisfaction” and the chain-mediating effect of “job autonomy → self-efficacy → work to family enrichment → family satisfaction” was significant. Thus, Hypothesis 3-2 and Hypothesis 4-2 were supported.

**TABLE 4:** Path analysis of job autonomy on family satisfaction.

Path relationship		Indirect effect of standardization	95% Confidence interval	
			Lowerbound	Upperbound
Direct effect	JA→FS	0.31	0.14	0.61
Mediating effect	JA→WFE→FS	0.34 × 0.44 = 0.15	0.05	0.37
	JA→SE→WFE→FS	0.71 × 0.32 × 0.44 = 0.1	0.05	0.26
Total mediating effect		0.34 × 0.44 + 0.71 × 0.32 × 0.44 = 0.25	0.16	0.49
Total effect		0.31 + 0.25 = 0.56	0.51	0.86

*JA, job autonomy; WFE, work to family enrichment; FS, family satisfaction; SE, self-efficacy.*

### Hypothesis Testing

In the previous part, the data were processed by software, and the hypothesis relationship was tested. In the following part, the conclusion of each hypothesis test is summarized, as shown in Table 5.

**TABLE 5:** Test results of hypothesis.

Hypothesis		Test results
H1-1	The job autonomy of knowledge employees is positively correlated with job satisfaction.	Supported
H1-2	The job autonomy of knowledge employees is positively correlated with family satisfaction.	Supported
H2-1	The job autonomy of knowledge employees has a positive impact on job satisfaction through self-efficacy.	Not supported
H2-2	The job autonomy of knowledge employees has a positive impact on family satisfaction through self-efficacy.	Not supported
H3-1	The job autonomy of knowledge employees has a positive effect on job satisfaction through work-family enrichment.	Supported
H3-2	The job autonomy of knowledge employees has a positive effect on family satisfaction through work-family enrichment.	Supported
H4-1	The job autonomy of knowledge employees has a positive effect on job satisfaction through the chain mediating effect of self-efficacy and work-family enrichment.	Supported
H4-2	The job autonomy of knowledge employees has a positive effect on family satisfaction through the chain mediating effect of self-efficacy and work-family enrichment.	Supported

### Path Analysis Among Variables in Modified Model 1

The empirical data support the hypothesis that job autonomy has a positive impact on job satisfaction of knowledge employees proposed in the modified Model 1. And in the two hypotheses of self-efficacy and work-family enrichment as mediating variables of job autonomy and job satisfaction, the former was not supported, but the latter was supported by the empirical data. The empirical data support the chain-mediating effect of job autonomy on job satisfaction through self-efficacy and then work-family enrichment.

Firstly, in the analysis of the relationship between job autonomy and job satisfaction, data processing results showed that job autonomy had a positive impact on job satisfaction, that is, the higher the work autonomy of knowledge employees, the higher the job satisfaction. Hypothesis 1-1 was supported. The results were consistent with the COR theory that “the more abundant the resources, the easier it is for individuals to obtain the resources,” which indicates that improving the job autonomy of knowledge employees can accurately improve their job satisfaction.

Secondly, the results pointed out that job autonomy had a positive effect on self-efficacy, which was consistent with the research results of Hobfoll and Shirom (2001) and Zhang et al. (2010). This proves that the higher the job autonomy of knowledge employees, the higher their self-efficacy. However, the mediating effect of self-efficacy on job autonomy and job satisfaction was not significant; and, as such, Hypothesis 2-1 had not been supported. Previous studies have shown that psychological capital is an important mediating variable between the work domain and the family domain (Liu et al., 2018). However, because psychological capital is composed of optimism, self-efficacy, hope, and resilience, the mediating effect of psychological capital is greater than that of a single factor (Luthans et al., 2007). Self-efficacy does not fully represent psychological capital so that the mediating effect is not significant (Aziz et al., 2018).

Thirdly, the results showed that, in Hypothesis 3-1, the job autonomy of knowledge employees had a positive impact on job satisfaction through the work-family enrichment path, and the job autonomy had a positive impact on the work-family enrichment, which indicates that knowledge employees can make full use of the resources in the work domain to achieve the cross-border enrichment path, and the more work-family enrichment they will have. The results also showed that work to family enrichment had a positive impact on job satisfaction. That means the level of work to family enrichment directly determines the job satisfaction of knowledge employees, which has a very important impact on enterprise managers, and can be used as a new perspective to motivate knowledge employees. In addition, the results showed that work-family enrichment played a mediating role between job autonomy and job satisfaction, which confirmed the previous research (Zhang et al., 2012; Daniel and Sonnentag, 2016); thus, Hypothesis 3-1 was supported. This indicates that job autonomy has a direct impact on job satisfaction, and part of the impact is realized through the work to family enrichment.

Fourthly, the chain-mediating effect of self-efficacy and work to family enrichment on the relationship between job autonomy and job satisfaction of knowledge employees showed that self-efficacy and work to family enrichment have a significant chain-mediating effect on the main effect between job autonomy and job satisfaction, thereby giving support for Hypothesis 4-1. The results showed that the job autonomy of knowledge employees first affected their inner self-efficacy, and then improved their job satisfaction through the path of work to family enrichment. When knowledge employees have a high level of job autonomy, they tend to have a greater sense of self-confidence and self-efficacy in completing work tasks, and come up with more solutions so as to view the relationship between work and family from a more positive point of view, and then more flexibly assume the important task of work, improve the achievements in the field of work, and ultimately improve job satisfaction.

### Path Analysis Among Variables in Modified Model 2

The hypothesis that job autonomy has a positive impact on family satisfaction of knowledge employees proposed by the modified Model 2 was supported by the empirical data. Self-efficacy and work-family enrichment are two hypotheses of mediating variables of job autonomy and family satisfaction, respectively. The former was not supported, while the latter was supported by the empirical data. Job autonomy first affected family satisfaction through self-efficacy, and then through the path of work to family enrichment, which was supported by the empirical data.

Firstly, in the analysis of the relationship between job autonomy and family satisfaction, data processing results showed that the relationship between job autonomy and family satisfaction was significant, giving support for Hypothesis 1-2. That is to say, the higher the job autonomy of knowledge employees, the stronger their work control, which is conducive to the accumulation of more work resources, such as time, communication skills, salary, happy mood, and so on. These work resources will be put into the family field to improve the performance of employees in the family domain, and the family will give more positive feedback and, ultimately, improve family satisfaction.

Secondly, the mediating effect of self-efficacy of knowledge employees on job autonomy and family satisfaction was not significant; thus, Hypothesis 2-2 had not been supported. In addition to the fact that self-efficacy is less effective than psychological capital in predicting satisfaction, Bandura et al. (1999) also pointed out that general self-efficacy is less effective than self-efficacy in specific areas in predicting individual performance in specific areas. Therefore, the definition of self-efficacy is too broad, which reduces the correlation between self-efficacy and satisfaction in specific areas of work and family.

Thirdly, the mediating effect of work to family enrichment on job autonomy and family satisfaction of knowledge employees was significant, that is, the higher the job autonomy perceived by knowledge employees, the stronger the tendency of work to family enrichment, and family satisfaction also increases. Therefore, Hypothesis 3-2 was supported. This result showed that job autonomy can promote the improvement of family satisfaction through work to family enrichment. Knowledge employees can gain more autonomy in their work field, arrange more suitable work procedures, work progress and work methods, improve work efficiency and positive emotions, put the saved time and positive emotions into their families, have more positive interaction with their families, and, ultimately, improve their family satisfaction. It is supported that the continuous acquisition of resources mentioned in the COR theory is easy to form a gain spiral. So, the job autonomy of knowledge employees has been accumulated in the work domain and applied to the family domain to form positive results, which can predict that more family resources will be generated in the family domain, and these resources have a good incentive effect on the motivation of employees to fully participate in the role and put in their own efforts (Lazarova et al., 2010; Christian et al., 2011), thus showing higher family satisfaction.

Fourthly, the results showed that self-efficacy and work to family enrichment have a significant chain-mediating effect between job autonomy and family satisfaction, giving support for Hypothesis 4-2. The results proved that the specific path of job autonomy of knowledge employees affecting family satisfaction is through self-efficacy, and then through the work to the family enrichment path, and finally improve their family satisfaction. When knowledge employees have a high level of job autonomy, they tend to have a greater sense of self-confidence and self-efficacy in completing tasks in the family domain, and come up with more solutions so as to view the relationship between work and family from a more positive point of view, and then more flexibly assume the responsibilities of the family, improve the achievements in the family domain, and, ultimately, improve family satisfaction.

### Comparison of Two Models

There are some similarities and differences in the results of modified Model 1 and modified Model 2. The similarities are as follows: Firstly, in the structural equation model, the path coefficients of self-efficacy and job satisfaction, self-efficacy, and family satisfaction were not significant, so the two paths were deleted. Secondly, the main effects of the two models were significant, that is, job autonomy was positively correlated with job satisfaction, and job autonomy was positively correlated with family satisfaction; the mediating effect was significant, that is, job autonomy had a positive impact on job satisfaction through work to the family enrichment path, and job autonomy had a positive impact on family satisfaction through work to the family enrichment path; the chain mediating effect was significant, that is, job autonomy had a positive impact on job satisfaction through self-efficacy and work to family enrichment, and job autonomy had a positive impact on family satisfaction through self-efficacy and work to family enrichment. In comparison, the differences are as follows: Firstly, the main effect of modified Model 1 was slightly greater than that of modified Model 2, that is, the effect of “job autonomy → job satisfaction” (0.39^**^) was slightly greater than that of “job autonomy → family satisfaction” (0.24^**^). Secondly, the mediating effect of modified Model 1 was greater than that of modified Model 2, that is, the mediating effect of “job autonomy → work to family enrichment→ job satisfaction” (0.19) was greater than that of “job autonomy → work to family enrichment→ family satisfaction” (0.15). Finally, the chain-mediating effect of modified Model 1 was slightly greater than that of modified Model 2, that is, the chain-mediating effect of “job autonomy → self-efficacy → work to family enrichment→ job satisfaction” (0.12) was slightly greater than that of “job autonomy → self-efficacy → work to family enrichment→ family satisfaction” (0.1).

## Discussion

From the perspective of work-family enrichment, combined with self-efficacy perception in positive psychology, this study aims to explore the specific path of job autonomy affecting employee satisfaction through self-efficacy perception and work-family enrichment. It is found that job autonomy improves the self-efficacy of knowledge employees, and then improves their overall satisfaction through the path of work to family enrichment (that is, the establishment of the chain intermediary mechanism of “job autonomy – self-efficacy – work to family enrichment – job satisfaction” and “job autonomy – self-efficacy – work to family enrichment – family satisfaction”). At the same time, through comparison, it is found that job autonomy is more closely related to the positive emotions in the resource contribution domain (work). The main contributions of this study are summed as follows:

### Theoretical Implications

Firstly, this study explored the black box between environmental resources and work-family enrichment, that is, self-efficacy. Previous studies have found that job autonomy has a positive impact on work-family enrichment (Grzywacz and Marks, 2000; Demerouti and Geurts, 2004; Voydanoff, 2004). Therefore, it is reasonable to believe that, for knowledge employees, job autonomy, as an important environmental resource, will play an important role in their work-family enrichment. While the influence process of external factors on people is stimulation-psychology behavior, knowledge employees are the carriers of role resources and the subject of multiple role participation. Their individual perception evaluations and psychological factors often fluctuate with the changes of resources and individual characteristics provided by the organization. Therefore, based on COR and RGD theory, the current study selected self-efficacy as a mediating variable to explore the mediating effect of self-efficacy between job autonomy and work to family enrichment, trying to enrich the specific mechanism of environmental resources and work-family enrichment.

Secondly, the current study enriches the outcome variables of the family domain in work-family enrichment research. Although some scholars have pointed out that the relationship between work-family enrichment and family variables is the focus of future research, it is related to whether the contribution domain of resources is more closely related to the contribution domain (work) or the acceptance domain (family) (Wayne et al., 2006, 2007). It is found that work-family enrichment is more closely related to the contribution domain (work).

Thirdly, the study enriches the research methods of improving the satisfaction of knowledge employees. In social science research, mediation is one of the important methodological concepts. Mediation research can help us explain the mechanism between independent variables and dependent variables, and sort out the relationship between multiple variables. Previous studies mostly established the simple intermediary model and the multistep intermediary model to explore the improvement of employee satisfaction, which ignored the specific internal mechanism between variables to a certain extent. Because of the relationship between self-efficacy and work-family enrichment, this study established a chain mediation model to explore the specific mechanism of improving satisfaction. The chain mediation model innovatively combines the far-end variable (job autonomy) and the near-end variable (self-efficacy) that affect the satisfaction, and better reveals the internal and external factors that affect the satisfaction of knowledge employees.

### Practical Implications

Not only do the needs of different types of employees differ, but also the diversification of public needs has become a popular trend. Enterprises should cater to this trend by providing employees with incentive measures to meet their needs as much as possible, and then establish an incentive mechanism to meet their individual needs. On the one hand, enterprise managers should provide more resources or opportunities to meet the special needs of knowledge employees, such as job flexibility, professional technology and professional knowledge training, and educational opportunities. This is also in line with the COR theory that initial resources can create more resource accumulation and income for the future life of individuals, which is conducive to entering the gain spiral (Hobfoll, 1989). This will help improve the satisfaction of knowledge employees, thus forming a virtuous circle of accumulated resources in the domains of work and family. On the other hand, the contribution domain can produce positive effects on both the contribution domain and the acceptance domain through enrichment. Enterprises can give more job autonomy that meets the needs of knowledge employees as initial resources, which is conducive to employees to create more resources and benefits (such as satisfaction) in the field of work and family, forming a gain spiral in the two fields. In order to realize the development and value added of self-efficacy, the content of improving psychological capital should be integrated into the training programs of knowledge employees. In the past, the training contents mainly focused on the improvement of professional knowledge and skills of knowledge employees but ignored the cultivation of emotion, attitude, and values. In the process of training, the psychological capital intervention model (PCI model), which was created by Luthans and others, can be used to implement the intervention measures related to self-efficacy. Professionals can be invited to intervene in the training or lecture. Micro intervention is a short-term and highly focused form of development, which can significantly improve self-efficacy, job satisfaction, and job performance (Gilbert et al., 2018; Peeters et al., 2020).

The satisfaction of knowledge employees is mainly reflected through the psychological and physiological aspects of the actual satisfaction degree of each index in the work process of the enterprise. The performance of different satisfaction levels will affect the working status and ability of employees in the future. In this regard, the human resources management departments of enterprises need to establish a robust scientific satisfaction evaluation system for knowledge employees to reflect their satisfaction status in time. As an important part of the competitiveness of modern enterprises, the knowledge innovation ability of knowledge employees is an important driving force for the development of enterprises. In the specific operation process, anonymous evaluation is adopted to reduce the psychological burden of employees when they participate in evaluation activities. At the same time, this can truly reflect the exact captured ideas of knowledge employees for the development of the enterprise. Therefore, the individuals and personnel in related fields need to pay full attention to the satisfaction of knowledge employees by efficiently and effectively understanding and mastering the work status and development needs of knowledge employees. In addition, enterprises can also take the dimensions of personal growth and development system, salary and welfare of employees, and work relationship closely related to the life of knowledge employees, as the basic evaluation indicators to deeply understand the attitudes of knowledge employees.

Knowledge employees should strive for job autonomy through different ways. For example, they should actively participate in all kinds of social activities, maintain a good harmonious relationship with the enterprise managers and colleagues through such activities, and strive for more job autonomy for themselves (Chen et al., 2018). Furthermore, employees should also learn how to use and transform these work resources, improve the performance skills of personal work role and family role, and maximize the work-family enrichment.

Bandura said in his theory of self-efficacy that the level of individual self-efficacy will increase with the number of times to achieve their goals. Therefore, employees should set appropriate goals for themselves. Whenever the goals are completed, the sense of self-efficacy will be enhanced, thus forming a virtuous circle. At the same time, physical state and psychological state are the key factors to construct self-efficacy. A more stable psychological state and higher psychological maturity are very important for knowledge employees who have been under high pressure and intensity for a long time, which can help them overcome problems and setbacks in different domains. Therefore, it is paramount for knowledge employees to learn to adjust themselves actively, balance their psychology, and maintain a state of physical and mental harmony.

### Limitations and Future Directions

No study is immune to, and without, limitations, and this study, too, is no exception. This study has several limitations and possible future directions.

Firstly, the control of demographic variables was not strict enough. The current study explored the effect of improving satisfaction of knowledge employees from the perspective of work-family enrichment. However, in order to ensure sufficient sample size in data analysis, some compromises were made on the basis of previous studies. For example, it only limited the educational background and occupation categories, and did not control other variables that may have had an impact, such as working years and marital status. The lax control of demographic variables may have affected the reliability of the results.

Secondly, all variables were self-reported. The data of each variable in the study were derived from self-reports of employees, ignoring the feedback from other members of the organization and family. The feedback of family members, colleagues, and leaders on the variables of knowledge employees are more objective and different from the self-evaluation of employees and, as such, can reflect the situation and circumstances of employees more justly and truthfully without bias (Bhave and Lefter, 2018). Therefore, any follow-up research should adopt the method of multi-evaluation to measure the work-family interface variables of employees.

Thirdly, cross-sectional data cannot show causality. Due to the limitation of time and funds, cross-sectional data were adopted in the current study, and no longitudinal data reflecting causality were adopted. Cross-section data can only reflect the observed value of a general characteristic variable, which is easy to cause the problem of multi-collinearity. In addition, the questionnaire is easily affected by accidental factors, such as mood, social approval, and other factors, which will affect the data quality and the authenticity of research results. In the future empirical study of work-family enrichment, a longer survey cycle should be planned to obtain longitudinal data to explore the causal relationship of work-family enrichment.

It is suggested that future researchers can extend the research cycle and adopt a longitudinal research design to help explain the causality of work-family enrichment, pay attention to the change of work to family enrichment and the family to work enrichment path after COVID-19, and enrich the exploration of outcome variables in the family domain. In addition, cross-level research, team-level research and cross-country cultural comparative research are also worth exploring to verify the similarities and differences of existing theories at different levels.

## Conclusion

The current study concludes that job autonomy of knowledge employees positively promotes job satisfaction and family satisfaction. This study also confirms that self-efficacy, together with work to family enrichment, plays a chain-mediating role in the main effect. In summation, this study is conducive to help enterprises invest work resources more accurately and effectively to obtain higher satisfaction of knowledge employees, improve the stability and enthusiasm of key personnel, and provide a new perspective for the stable development of enterprises and employees.

## Data Availability Statement

The original contributions presented in the study are included in the article/supplementary material, further inquiries can be directed to the corresponding author.

## Ethics Statement

Ethical review and approval was not required for the study on human participants in accordance with the local legislation and institutional requirements. Written informed consent for participation was not required for this study in accordance with the national legislation and the institutional requirements. The patients/participants provided their oral informed consent to participate in this study.

## Author Contributions

SJ and ZL were responsible for the conception and the design of the study and organized the database. XG wrote the English draft of the manuscript. DS revised the English version. All authors read and approved the final submitted version.

## Conflict of Interest

The authors declare that the research was conducted in the absence of any commercial or financial relationships that could be construed as a potential conflict of interest.

## Publisher’s Note

All claims expressed in this article are solely those of the authors and do not necessarily represent those of their affiliated organizations, or those of the publisher, the editors and the reviewers. Any product that may be evaluated in this article, or claim that may be made by its manufacturer, is not guaranteed or endorsed by the publisher.
